# m^6^A reader proteins: the executive factors in modulating viral replication and host immune response

**DOI:** 10.3389/fcimb.2023.1151069

**Published:** 2023-05-30

**Authors:** Decheng Yang, Guangze Zhao, Huifang Mary Zhang

**Affiliations:** ^1^ Department of Pathology and Laboratory Medicine, University of British Columbia, Vancouver, BC, Canada; ^2^ Centre for Heart Lung Innovation, St. Paul’s Hospital, Vancouver, BC, Canada

**Keywords:** N^6^-methyl adenosine, m^6^A reader, epitranscriptomics, viral replication, immune response

## Abstract

N^6^-Methyladenosine (m^6^A) modification is the most abundant covalent modification of RNA. It is a reversible and dynamic process induced by various cellular stresses including viral infection. Many m^6^A methylations have been discovered, including on the genome of RNA viruses and on RNA transcripts of DNA viruses, and these methylations play a positive or negative role on the viral life cycle depending on the viral species. The m^6^A machinery, including the writer, eraser, and reader proteins, achieves its gene regulatory role by functioning in an orchestrated manner. Notably, data suggest that the biological effects of m^6^A on target mRNAs predominantly depend on the recognition and binding of different m^6^A readers. These readers include, but are not limited to, the YT521-B homology (YTH) domain family, heterogeneous nuclear ribonucleoproteins (HNRNPs), insulin-like growth factor 2 mRNA-binding proteins (IGF2BPs), and many others discovered recently. Indeed, m^6^A readers have been recognized not only as regulators of RNA metabolism but also as participants in a variety of biological processes, although some of these reported roles are still controversial. Here, we will summarize the recent advances in the discovery, classification, and functional characterization of m^6^A reader proteins, particularly focusing on their roles and mechanisms of action in RNA metabolism, gene expression, and viral replication. In addition, we also briefly discuss the m^6^A-associated host immune responses in viral infection.

## Introduction

Structural and functional analyses of RNA modifications have recently become a hot area of epigenetic research. In eukaryotes, posttranslational modification of RNA is very common and over 100 types of covalent modifications have been discovered so far (Boccaletto et al., 2018). Among them, the most prevalent modification is methylation at the N^6^ of adenosine. The N^6^-methyladenosine (m^6^A) modification was discovered early in mRNAs. With advances in molecular detection techniques, m^6^A modifications have been increasingly discovered in many other types of RNAs, such as mRNAs, microRNAs, long non-coding RNAs, and circular RNAs (Chen et al., 2019; Zaccara et al., 2019). Sequence analyses have found that, in mRNAs, ~0.1%–0.4% of adenosines are N6-methylated (Wei et al., 1975; Sommer et al., 1978) and ~25% of cellular mRNAs contain multiple m^6^A residues (Wei and Moss, 1977; Csepany et al., 1990). Epitranscriptomic analyses have found that m^6^A sites are highly conserved and generally enriched in the consensus motif RRACH (R= G or A, and H = A, C, or U), which is more likely to be detected in the 3′-untranslated regions (3′UTRs), near stop codons and within internal long exons (Dominissini et al., 2012; Meyer et al., 2012). The presence of a long internal exon, which is defined as an exon that is much longer than the ~140-bp length of a typical exon, is considered to be an inducer of m^6^A deposition within a transcript (Dominissini et al., 2012; Ke et al., 2015; Ke et al., 2017).

The m^6^A level of an mRNA is dynamically maintained at a relatively balanced level *via* the crosstalk of several enzymes, including methyltransferases (writers) and demethylases (erasers). For example, in HIV and Enterovirus infection, viral infection can increase writer (METTL3/14 (methyltransferase 3/14)) and decrease eraser (FTO (fat mass and obesity-associated protein) or ALKBH5 (alkB homolog 5)) levels and thus promote viral replication. This dynamic and functional relevance of m^6^A for viral replication was further verified by silencing of the m^6^A writer or eraser enzymes, which decreased and increased viral replication, respectively (Lichinchi G et al., Nat Microbiol. 1: 16011, 2016; Hao H et al., Nucleic Acids Res. 47: 362, 2018). METTL3, a core component with methyltransferase activity, can combine with S-adenosylmethionine (SAM) to catalyze RNA methylation in the nucleus during transcription (Liu et al., 2014; Wang et al., 2016). However, METTL14 is an allosteric activator and functions as an RNA-binding platform to form a stable heterodimer with METTL3, strengthening the catalytic effect of METTL3 (Liu et al., 2014; Wang et al., 2016). Interestingly, WTAP (WT1-associated protein) interacts with the METTL3–METTL14 complex to ensure its localization to nuclear speckles and also modulates its recruitment to mRNA targets to promote its catalytic activity (Ping et al., 2014). Some other catalytic subunits or regulatory factors, such as vir-like m^6^A methyltransferase associated (VIRMA, also termed KIAA1429), RNA-binding motif protein 15/15B (RBM15/RBM15B), HAKAI (also named CBLL1, Cbl proto-oncogene-like 1), and zinc finger CCCH domain-containing protein 13 (ZC3H13), have also been described. VIRMA preferentially mediates m^6^A modification in the 3′UTR and near the stop codon, affecting the selection of methylation sites (Yue et al., 2018). RBM15/RBM15B plays an important role in X-inactivation and gene silencing *via* m^6^A modification of long non-coding RNA XIST (X-inactive specific transcript) (Patil et al., 2016). HAKAI, an E3 ubiquitin-protein ligase, functions as an associated component of the WTAP complex in the induction of m^6^A methylation (Yue et al., 2018). ZC3H13 induces the nuclear localization of the Zc3h13–WTAP–Virilizer–Hakai complex to regulate m^6^A modification (Wen et al., 2018). Recently, some novel methyltransferase accessory factors have been discovered. For example, methyltransferase ZCCHC4 (zinc finger CCHC-type containing 4) is responsible for the m^6^A methylation of 28S rRNA, while METTL5 (methyltransferase 5) can modify the N^6^-adenosine of 18S rRNA with the assistance of TRMT112 (TRNA methyltransferase activator subunit 11-2), an allosteric activator facilitating the binding of the catalytic complex to the RNA target (Ma et al., 2019; Ren et al., 2019; van Tran et al., 2019). Another enzyme is METTL16 (methyltransferase 16). It catalyzes the formation of a single m^6^A in the U6 small nuclear RNA (snRNA) involved in splicing (Pendleton et al., 2017). Additionally, METTL16 can catalyze the formation of m^6^A in a small number of other mRNAs and non-coding RNAs (Warda et al., 2017).

m^6^A modifications can be removed by RNA demethylases. Currently, only two demethylases have been identified and both are AlkB family proteins. The first one is FTO, which is located in the nucleus and abrogates m^6^A levels *via* oxidative demethylation activity (Jia et al., 2011). The second is the ALKBH5, which can remove the m^6^A modification on nuclear RNAs and further modulate nuclear RNA export and RNA metabolism (Zheng et al., 2013). Functional studies have found that FTO is responsible for gaining body weight when having mutations (Dina et al., 2007; Frayling et al., 2007; Do et al., 2008; Jia et al., 2011), and ALKBH5 was reported to be important for mouse fertility as mice deficient in ALKBH5 have shrunken testis (Zheng et al., 2013). More recent studies have suggested that FTO and ALKBH5 have different substrate specificities. ALKBH5 demethylates transcripts containing m^6^A, including viral transcripts (Zheng et al., 2013; Lichinchi et al., 2016b). However, FTO has greater substrate specificity not only for mRNAs containing m^6^A but also for the 5′ cap-adjacent nucleotide containing m^6^A, 2′*O*-methylation (m^6^Am), which occurs on about 10% of transcripts (Mauer et al., 2017).

**Figure 1:**
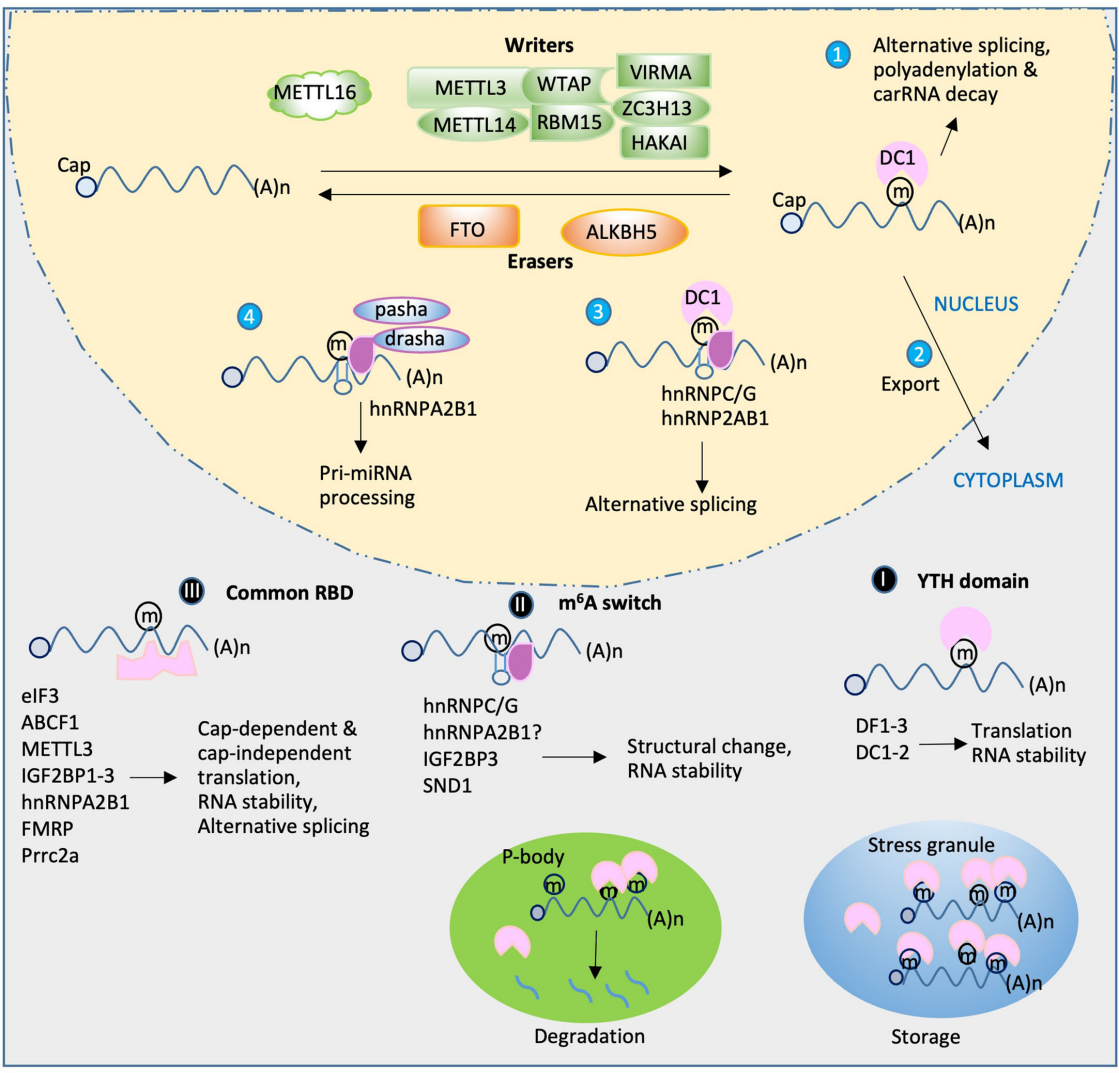
Functions of m^6^A reader proteins. RNA transcripts are m^6^A-methylated by a m^6^A writer complex composed of core subunits METTL3 and METT14 and additional accessory factors. METTL16 installs m^6^A on U6 snRNA and *MAT2A* mRNA. The m^6^A can be removed by demethylase FTO or ALKBH5. The reader proteins (purple) play diverse roles in RNA metabolism and gene expression. In nucleus, DC1 plays a role in alterative splicing, polyadenylation and carRNA decay (1) as well as m^6^A RNA export (2). The other readers hnRNPC/G or hnRNP2AB1 mediates alternative splicing (3) and pri-miRNA processing when drasha and pasha present (4). After export of the m^6^A RNA, three classes of readers in cytoplasm bind the m^6^A RNA in different mechanisms: (I) YTH readers bind m^6^A directly and regulate translation and RNA stability; (II) m^6^A switch-mediated binding. A local hairpin structure disrupted by m^6^A methylation favors binding events of a group of readers to regulate RNA structural changes and stability; (III) binding through tandem common RNA binding domain (RBD), such as KH, RGG and GRE domain. These readers as indicated regulate translation, splicing and RNA stability. During cellular stress, certain multiple m^6^A-containing RNAs and readers facilitate phase-separated compartment formation to produce stress granules for storage or transfer to P-body, leading to degradation.

Although the m^6^A writer and eraser play important roles in m^6^A-mediated gene regulation, the effects of m^6^A modification on RNA metabolism, gene regulation, and many other biological functions predominantly depend on m^6^A recognition by different m^6^A-binding proteins (also called “readers”). The known reader proteins include, but are not limited to, the YT521-B homology (YTH) domain family, heterogeneous nuclear ribonucleoproteins (HNRNPs), and insulin-like growth factor 2 mRNA-binding proteins (IGF2BPs). Several studies have demonstrated that these proteins are not only involved in normal gene regulation but also play crucial roles in modulating the pathophysiological conditions by interacting with the target mRNAs (**Figure 1**). In this review, we focus on recent advances in the discovery, classification, and functional characterization of m^6^A reader proteins, particularly focusing on their roles and mechanisms in RNA metabolism, gene regulation, and viral replication. In addition, we also briefly discuss the m^6^A-associated host immune responses in viral infection.

## Classification of m^6^A readers

m^6^A reader proteins, as the crucial factors in regulating m^6^A-modified transcripts, have been identified in many studies. The YTH domain-containing m^6^A readers (YTH readers) were the first discovered group of m^6^A readers and provided a mechanism for understanding the effects of m^6^A on mRNA biology (Dominissini et al., 2012). m^6^A methylation can destabilize the mRNA structure, which can affect the binding of diverse RNA-binding proteins (potential readers), leading to altered translation efficiency or other functional changes. With the technical advances in mapping of m^6^A sites and identifying m^6^A-binding proteins within the transcriptome, an increasing number of m^6^A readers have been discovered. Here, we summarize some of the major discoveries of m^6^A readers and their cellular functions, although controversies still exist on their functions among different reports.

## Direct m^6^A readers

### Direct m^6^A readers that contain a YTH domain

The direct m^6^A reader group has five YTH domain-containing members, namely, three YTHDF1-3 (DF1-3) and two YTHDC1-2 (DC1-2) readers. Hereafter, we call these five proteins YTH readers. These proteins bind mRNA in an m^6^A-dependent manner. The YTH readers are evolutionarily conserved and independent of cell type (Wang et al., 2017). The cellular localization of these proteins differs: DF1-3 are cytosolic (Wang et al., 2014; Li et al., 2017a; Shi et al., 2017), DC1 is predominantly nuclear (Hartmann et al., 1999), and DC2 can be both nuclear and cytosolic (Wojtas et al., 2017). Structural studies have demonstrated that the selectivity of YTH readers for binding the methyl moiety of m^6^A is achieved mainly *via* a “tryptophan cage” (Luo and Tong, 2014; Xu et al., 2014; Xu et al., 2015), in which two or three tryptophans wrap around the methyl group. The three members (DF1–3) of the DF family proteins share high similarity in their amino acid (aa) sequence. In addition to the YTH domain (100–150 aa) of the protein, the remaining ~400-aa region contains several prion-like P/Q/N-rich domains (Li et al., 2014; Zhu et al., 2014) that can cause DF proteins to undergo “liquid-liquid phase separation” (LLPS). This LLPS is markedly enhanced by mRNAs that contain multiple, but not single, m^6^A residues. The resulting m^6^A-rich lipid droplets then participate in the formation of phase-separated compartments, such as stress granule and P-body (Ries et al., 2019) (**Figure 1**). From this point of view, PLrD may play a role in storage/protection of m^6^A-containing mRNA during the methylation process. Furthermore, the produced phase-separated compartments may negatively regulate certain viral replication by blocking viral particle assembly, such as that in Flavoviruses (Gokhale,N et al., Cell Host & Microbe, 20: 654, 2016). In addition, stress granule formation during viral infection is an antiviral response since stress granule is a platform of antiviral type I interferon immune signaling (Onomoto et al., 2012). However, a recent study showed that m^6^A modifications only play a minimal, or even no, role in mRNA partitioning into stress granules (Khong et al., 2022).

Although all three DF (DF1–3) proteins can enhance m^6^A-mRNA phase separation, there is conflicting evidence about whether they each have specialized effects on m^6^A-modified mRNAs. Earlier reports found that each of the three DF proteins has a different effect on m^6^A mRNAs; for instance, DF1 promotes the translation of m^6^A-modified mRNAs through interaction with the translation initiation factor eIF3 (eukaryotic translation initiation factor 3) rather than with the m7G-cap structure (Wang et al., 2015), DF2 enhances m^6^A mRNA degradation by recruiting m^6^A mRNAs to decay sites (Wang et al., 2014), and DF3 not only enhances translation by interacting with DF1 but also promotes degradation by associating with DF2 (Wang et al., 2014; Shi et al., 2017; Patil et al., 2018). However, other studies demonstrated that all three DFs have similar roles in mRNA degradation by recruiting the carbon catabolite repression 4 (CCR4)—negative on the TATA-less (NOT) deadenylation complex to m^6^A mRNAs (Du et al., 2016; Kennedy et al., 2016). Further studies on the crosstalk of these three DF proteins found that, remarkably, DF3 can facilitate mRNA translation of DF1/3 common targets, but not DF3 unique targets. Intriguingly, DF3 depletion decreases the binding of DF1 and DF2 to their target transcripts, while DF1 or DF2 loss reduces the amount of RNA that is bound by DF3 (Shi et al., 2017). Given the high sequence similarity among the three DF proteins, it is unclear how DFs mediate different functions. Another issue is whether DF proteins can bind different or the same m^6^A sites in mRNAs. Some recent studies have suggested that most m^6^A residues only bind one of the three DF paralogues (Shi et al., 2017), whereas others have suggested that all m^6^A sites bind all DF paralogues in largely equivalent manners (Patil et al., 2016). Since the m^6^A sites are largely located in the 3′UTR, stop codon region, the 5′UTR, and sometimes the coding sequences themselves (Dominissini et al., 2012; Meyer et al., 2012), binding of the reader to different m^6^A sites may mediate distinct functions. Additional studies are needed to clarify mechanisms of binding of DF proteins and their functional consequences.

As mentioned earlier, the three DFs and two YTHDCs have differential cellular localization. DC1 is usually found in the nucleus (Xu et al., 2014) and can bind mRNAs shortly after they are transcribed and methylated. Upon binding, DC1 may induce alternative splicing (i.e., promoting exon inclusion) by recruiting RNA splicing factor SRSF3 (serine and arginine-rich splicing factor 3) and blocking SRSF10 (serine and arginine-rich splicing factor 10) from binding to mRNAs (Xiao et al., 2016). DC1 also interacts with SRSF3 and NXF1 (nuclear RNA export factor 1) to facilitate m^6^A mRNA nuclear export (Roundtree et al., 2017). Furthermore, DC1 appears to mediate the function of m^6^A in non-coding RNA, such as XIST, a non-coding RNA that contributes to X chromosome inactivation and silencing of genes on the X chromosome (Patil et al., 2016). Another study on the non-coding chromosome-associated regulatory RNA (carRNA) found that m^6^A on carRNA facilitates transcriptional downregulation of proximal genes by inducing the decay of carRNA transcripts that regulate the chromatin state of proximal loci (Liu et al., 2020). DC2 is an RNA-induced ATPase with 3-to-5′ RNA helicase activity (Wojtas et al., 2017). Unlike other YTH domain-containing proteins, which are ubiquitously expressed, DC2 is enriched in the testes (Bailey et al., 2017; Hsu et al., 2017; Wojtas et al., 2017; Liu et al., 2021b). DC2 knockout mice show defects in spermatogenesis without other obvious developmental defects (Bailey et al., 2017; Hsu et al., 2017; Wojtas et al., 2017; Liu et al., 2021b). The binding properties of DC2 are unusual. It binds to m^6^A mRNA weaker than the four other YTH readers. Like other YTH proteins, the DC2 domain retains the “tryptophan cage” to bind methylated adenosine. However, the DC2’s YTH domain shows sequence discrepancy in the region that is predicted to bind m^6^A-adjacent residues (Patil et al., 2018). Transcriptome-wide mapping of DC2-binding sites by CLIP (CAP-Gly domain-containing linker protein 1) has shown low overlap with m^6^A sites (Patil et al., 2016). Thus, DC2 may bind select m^6^A sites or affect m^6^A mRNA through other binding mechanisms. Additionally, DC2 contains a helicase domain, R3H domain, and ankyrin repeats (Kretschmer et al., 2018), which may increase translation efficiency of target mRNAs by facilitating the interactions between m^6^A RNA and small subunit ribosomes (Hsu et al., 2017; Mao et al., 2019). Indeed, translation was increased when DC2 was artificially tethered to a reporter RNA (Hsu et al., 2017). Other studies suggest that DC2 mediates mRNA degradation through recruitment of the 5′–3′ exoribonuclease Xrn1 (Wojtas et al., 2017; Kretschmer et al., 2018) and thus decreases target gene expression.

### Direct m^6^A reader that lacks a YTH domain

The direct m^6^A readers that lack an YTH domain include eIF3, ATP-binding cassette F1 protein (ABCF1), METTL3, and heterogeneous nuclear ribonucleoproteins (HNRNPs). These readers preferentially bind m^6^A in the 5′UTR or non-coding regions. Although they can all bind m^6^A directly, HNRNPs seem to require m^6^A-induced RNA structural changes to facilitate binding.

#### eIF3 and related factors ABCF1 and METTL3

**Figure 2:**
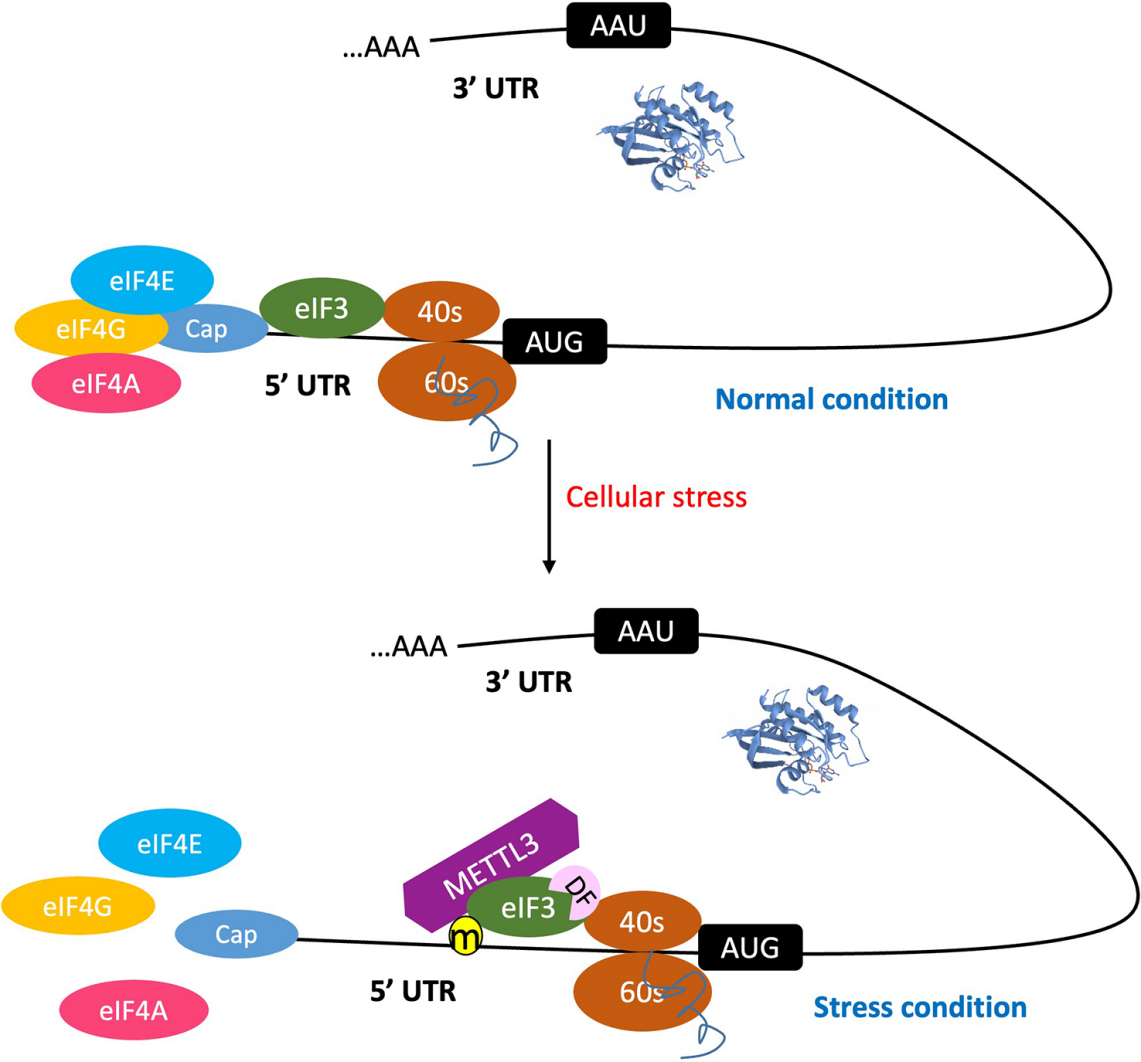
The 5'UTR m^6^A promotes cap-independent mRNA translation initiation. Under normal condition, the process of mRNA translation initiation occurs through a cap-dependent mechanism. When cells are subjected to stress, the m^6^A methylation machinery is activated. This leads to the destabilization of the mRNA secondary structure, thereby promoting the binding of reader protein eIF3 and/or METTL3 to the 5' UTR m^6^A site, and consequently initiating translation. This process is independent of the m^7^G-cap, as well as cap-binding proteins eIF4E and eIF4A. Furthermore, the reader DF-1 promotes translation by interacting with eIF3.

Since eIF3, ABCF1, and METTL3 can all bind the 5′UTR to participate in translation initiation control, these m^6^A readers can be discussed as a group (**Figure 1**). eIF3 is a multiprotein complex that functions during the initiation phase of eukaryotic translation (Aitken and Lorsch, 2012). Recent studies have demonstrated that m^6^A modifications in the 5′UTR, especially under cellular stress conditions (Coots et al., 2017; Perlegos et al., 2022), destabilize the local RNA secondary structure, facilitating the direct binding of reader eIF3 to initiate translation, independent of the m7G-cap and cap-binding protein eIF4E. The typical example of such is the translation of heat shock protein 70 (Hsp70) mRNA during heat shock condition (**Figure 2**). This pattern of translation initiation is incompatible with another cap-independent mechanism characterized by the requirement of an internal ribosome entry site (IRES) within the 5′UTR (Meyer et al., 2015).

Other potential m^6^A reader proteins involved in the regulation of cap-independent translation initiation are ABCF1 and METTL3. Recent studies revealed that ABCF1 plays a crucial role in cap-independent translation of m^6^A mRNAs. This notion is supported by the observation of selective translation of heat shock-induced protein 70 (Hsp70) mRNA during cellular stress (Coots et al., 2017). A further investigation of quantitative proteomic data revealed that eIF2a, eIF5, and ABCF1 are closely associated with stress-induced Hsp70 mRNA. ABCF1-mediated promotion of translation initiation of m^6^A mRNA is supported by data, demonstrating that in cells lacking ABCF1, heat shock-induced Hsp70 translation was severely impaired.

METTL3 is a nuclear enzyme responsible for adding m^6^A on transcripts and is thus usually recognized as a writer. However, a recent study discovered that METTL3 also has a cytosolic role in translation, acting as a “reader” rather than a “writer” of methylated transcripts as its m^6^A catalytic (writer) activity is dispensable in METTL3-promoted translation (Lin et al., 2016). Further, Coots et al. provided evidence to verify that METTL3 directly binds to internal m^6^A but not to the 5′ m7G cap of mRNA and that depletion of METTL3 selectively inhibits translation of mRNAs bearing 5′UTR methylation but not 5′TOP mRNA barely having m^6^A (Coots et al., 2017).

#### HNRNP family proteins—the direct readers requiring m^6^A switch

Unlike eIF3 and its related factors, the other three non-YTH m^6^A readers, namely, HNRNPC, HNRNPG, and HNRNPA2B1, have all been found in the nucleus. These readers convey the molecular roles of m^6^A on pre-mRNA splicing, mRNA stability, translation, and storage control as the YTH readers do (Alarcon et al., 2015a; Liu et al., 2015; Liu et al., 2017) (**Figure 1**). HNRNPC and HNRNPG preferentially bind m^6^A-modified RNAs *via* a “m^6^A-switch” mechanism in which the m^6^A modifications destabilize the RNA hairpin structure, leading to the exposure of single-stranded RNA that facilitates protein binding (Liu et al., 2015; Liu et al., 2017). Conversely, HNRNPA2B1 recognizes its m^6^A-modified targets not *via* an m^6^A-switch mechanism but *via* direct binding of the methylated RGAC consensus site (Alarcon et al., 2015a). The binding of HNRNPA2B1 to m^6^A mRNA facilitates microRNA biogenesis *via* recruiting the microprocessor complex (drosha and pasha) to the pri-miRNA (Alarcon et al., 2015a; Alarcon et al., 2015b). However, a recent study indicated that HNRNPA2B1 is an indirect m^6^A reader because it does not specifically recognize m^6^A-containing RNA *in vitro*. This study also found that very few m^6^A sites exhibit proximal HNRNPA2B1 binding *in vivo* (Wu et al., 2018).

Very recently, a new m^6^A reader SND1 (Staphylococcal nuclease domain-containing protein 1) was discovered. It can stimulate the lytic reactivation of Kaposi’s sarcoma-associated herpesvirus (KSHV) through binding to ORF50/RTA (Baquero-Perez et al., 2019). Deletion of SND1 leads to inhibition of KSHV early gene expression, showing that SND1 is essential for KSHV lytic replication.

## Indirect m^6^A readers

m^6^A mRNAs also indirectly recruit RNA-binding proteins. The known candidates include three insulin-like growth factor 2 mRNA binding proteins (IGF2BP1-3) and the fragile X messenger ribonucleoprotein (FMRP). Each of these RNA-binding proteins seems to enhance m^6^A mRNA stability (Edupuganti et al., 2017; Huang et al., 2018). IGF2BP1 relocalizes into mRNA aggregates in stress granules following heat stress, suggesting that it might also take part in the storage of translationally inactive mRNAs under stress conditions, thereby contributing to preventing their degradation (Huang et al., 2018). FMR1 negatively regulates the translation of a subset of m^6^A-modified transcripts, acting downstream of the m^6^A signal (Edupuganti et al., 2017).

Although both IGF2BPs and FMRP participate in the modulation of mRNA stability or translation efficiency in an m^6^A-dependent manner, their patterns that bind m^6^A mRNA directly or indirectly are not quite clear. Even though IGF2BPs may be direct m^6^A readers requiring an m^6^A structure switch (Sun et al., 2019), many other studies suggested that they are indirect m^6^A readers for the following reasons (Berlivet et al., 2019): first, several motif analyses of peaks by CLIP identified that FMRP and IGF2BP proteins bind a sequence motif that resembles the m^6^A consensus motif DRACH (Edupuganti et al., 2017; Huang et al., 2018); however, other CLIP studies suggested that IGF2BPs bind to a different consensus sequence. Second, the transcriptome-wide binding pattern determined by the distribution of their CLIP reads does not match the striking stop codon-enriched m^6^A distribution (Edupuganti et al., 2017; Huang et al., 2018). Third, in contrast to YTH domains, FMRP and IGF2BP proteins show weak binding affinities for m^6^A RNA and poor capacity to discriminate between these RNAs and non-methylated RNA (Edupuganti et al., 2017; Huang et al., 2018). Fourth, FMRP was found to directly bind reader DF2 (Youn et al., 2018; Zhang et al., 2018). Thus, FMRP may indirectly associate with m^6^A RNA through its interaction with DF proteins. Similarly, IGF2BP proteins interact with DF proteins in pulldown studies (Youn et al., 2018) and thus probably interact with m^6^A RNA indirectly. Recently, a proline-rich coiled-coil 2A (Prrc2a) protein was reported as a new reader, which specifically binds to a consensus GGm^6^ACU motif *via* a new Prrc2a domain (named GRE domain) to stabilize the critical transcript in neural cells (Wu et al., 2019). It is not clear whether Prrc2 in other organs contributes to the regulation of biological activities.

## Role of M^6^A reader proteins in animal viral infections

m^6^A readers not only play a critical role in normal and pathological conditions by interacting with cellular mRNAs but also are very important mediators that regulate viral infection by recognizing m^6^As on viral RNA. m^6^A modification of viral RNA was first discovered in nuclear viruses. These viruses are largely DNA viruses, such as simian virus 40 (SV40), adenovirus-2, and herpes simplex virus; m^6^A was later found in multiple nuclear RNA viruses such as retroviruses and influenza A virus (Lichinchi et al., 2016a; Courtney et al., 2017). Thus, early studies led the field to believe that the nucleus was the primary site of m^6^A modification of viral transcripts. Furthermore, as writer and eraser enzymes all reside in the nucleus at the steady state, it is reasonable to anticipate that any potential m^6^A modifications of viral RNAs would exclusively occur in the nucleus and would not apply to cytoplasmic RNA viruses. However, recent studies reported that the writer and eraser proteins can be detected in the cytoplasm of cells under stress conditions (Gokhale et al., 2016; Lichinchi et al., 2016b), indicating that these proteins can shuttle between the cytoplasm and the nucleus in response to stressors, such as viral infections. Thus, these discoveries raised the possibility that cytoplasmic viruses might also be m^6^A-modified. Indeed, in recent years, an increasing number of investigations have revealed that like DNA viruses, many RNA viruses, such as flaviviruses (e.g., hepatitis C virus (HCV), dengue virus and Zika virus (ZIKV)), enteroviruses, and coronaviruses, have m^6^A modifications on their viral genomic RNAs (Gokhale et al., 2016; Lichinchi et al., 2016b; Hao et al., 2019). These m^6^A methylations differentially regulate viral replication depending on the viral species (**Table 1**).

**Table 1:** Regulatory role of m^6^A reader proteins in viral replication.

Viral family	Virus	Reader protein	Relationship with virus	Impact on virus	Reference
	**RNA virus**				
*Flaviviridae*	ZIKV	YTHDF1-3	Bind to ZIKV m^6^A RNA	YTDHF1-3 expression suppress viral replication	Lichinchi et al., 2016b
	HCV	YTHDF1-3	Bind to m^6^A sites within the HCV E1 region	Decrease viral particle formation by suppressing viral RNA packaging	Gokhale et al., 2016
*Picornaviridae*	EV71	YTHDF1-3 YTHDC1		Knocking down YTHDF1-3 and YTHDC1 in AD cells enhances viral replication	Hao et al., 2019
	EV71	YTHDF2-3		Overexpression in Vero cells promotes viral replication	Hao et al., 2019
	PV/CVB3	YTHDF3	YTHDF1-3 are cleaved by enterovirus 2A^pro^	YTHDF3 inhibits viral replication by enhancing type I IFN signaling	Kastan et al., 2021
*Coronaviridae*	PEDV	YTHDF1-2	Bind to PEDV RNA in LLC-PK1 cells	Both inhibits viral replication; YTHDF2 reduces viral m^6^A RNA stability	Chen et al., 2020
	SARS-CoV2	YTHDF2		Negatively regulate viral infection	Liu et al., 2021a
*Togaviridae*	CHIKV	YTHDF1-2		YTHDF1 restricts viral replication; YTHDF2 promotes viral replication	Kim et al., 2020a
*Pneumoviridae*	RSV	YTHDF1-3		Increase viral protein expression, gRNA and mRNA synthesis and viral particle formation	Xue et al., 2019
*Paramyxoviridae*	HMPV	YTHDF1-3 YTHDC1		Promotes viral replication in both A549 and HeLa cells	Lu et al., 2020
*Rhabdoviridae*	VSV	YTHDF2 YTHDF3	Act as trans‐acting factors to inhibit RLRs from binding to m^6^A RNAs	YTHDF2 is essential for suppression of type I IFN responses; YTHDF3 is a negative regulator of antiviral immunity	Zhang et al., 2019b; Lu et al., 2021
*Reoviridae*	RV	Readers have not been studied		m^6^A modification has a negative effect on RV replication	Wang et al., 2022
*Orthomyxoviridae*	IAV	YTHDF1-3		YTHDF2 but not DF1 and DF3 significantly promotes IAV replication and viral particle production	Courtney et al., 2017
*Retroviridae*	HIV	YTHDF1-3	Bind to the m^6^A-modified leader sequence of viral RNA	Inhibits viral replication in HeLa cells	Tirumuru et al., 2016; Lu et al., 2018
	HIV	YTHDF1-3 YTHDF2		YTHDF1-3 boost HIV-1 protein and RNA expression in 393 cells; YTHDF2 promotes HIV replication in CEM-SS cells	Kennedy et al., 2016
	HIV	YTHDC1 YTHDF2 YTHDF3	YTHDC1 and YTHDF2 bind to multiple distinct and overlapping sites on the HIV-1 RNA; YTHDF3 is incorporated into HIV particles and cleaved by HIV protease.	YTHDC1 regulates the alternative splicing of viral RNAs and promotes viral infectivity; YTHDF2 increases the stability of viral RNAs; YTHDF3 reduces HIV infectivity	Jurczyszak et al., 2020; Tsai et al., 2021; N'Da Konan et al., 2022
	ERVs	YTHDF1-3		Suppress the intracisternal particle mRNA levels	Chelmicki et al., 2021
Viral family	Virus	Reader protein	Relationship with virus	Impact on virus	Reference
	DNA virus				
*Polyomaviridae*	SV40	YTHDF2-3		YTHDF2 but not YTHDF3 enhances viral particle formation	Tsai et al., 2018
*Hepadnaviridae*	HBV	YTHDF2-3	Bind to the pgRNA of HBV	Negatively regulate HBV pgRNA expression	Imam et al., 2018
	HBV	YTHDF2	Interact with viral m^6^A RNA; Form a complex with ISG20	Inhibits RIG-I-mediated immune response; Causes viral RNA degradation	Kim et al., 2020c; Imam et al., 2020
	HBV	YTHDC1 FMRP	Recognize m^6^A-methylated HBV transcripts and facilitate their transport to the cytoplasm	Benefits viral life cycle	Kim et al., 2021
*Adenoviridae*	AV	DC1 DF1 DF2		Do not affect the adenoviral infectious cycle; Reader expression level has no-change during AV infection but concentrates at sites of nascent viral RNA synthesis	Price et al., 2020
*Herpesviridae*	HSV-1	YTHDF1-3	HSV-1 infection enhances YTHDF1-3 expression at an early stage	Silencing YTHDF3 strikingly decreased viral replication	Srinivas et al., 2021; Feng et al., 2022
	HCMV	YTHDF2-3		YTHDF2 promotes HCMV replication by suppressing the induction of ISGs; YTHDF3 promotes HCMV propagation by enhancing turnover of T1IFN mRNAs	Rubio et al., 2018; Winkler et al., 2019
	EBV	YTHDF1		Suppress EBV infection and replication by enhancing viral m^6^A RNA degradation	Lang et al., 2019
	KSHV	YTHDC1 YTHDF2 YTHDC2 SND1	Targets viral ORF50/RTA mRNA during reactivation	YTHDC1 may or may not be involved in viral RTA pre-mRNA splicing in B cells; YTHDF2 destabilizes ORF50/RTA mRNA during reactivation in epithelial cells; YTHDF2 enhances ORF50/RTA expression, lytic reactivation and viral particle release in SLK.219 cells. YTHDC2 is essential for IL6 mRNA to escape from KSHV endoribonuclease SOX; SND1 is essential for KSHV lytic replication	Ye et al., 2017; Hesser et al., 2018 Hesser et al., 2018 Hesser et al., 2018; Tan et al., 2018 Baquero-Perez and Whitehouse, 2015 Baquero-Perez et al., 2019
***Baculoviridae***	**BmNPV**	**YTHDF3**	**Binds viral *ie-1* mRNA**	**Inhibits viral replication**	Zhang et al., 2022

## m^6^A and its reader proteins in RNA virus infection

### Flaviviruses

The flaviviruses are positive single-stranded RNA (ssRNA) viruses that replicate in the cytoplasm. Two laboratories have mapped m^6^A methylation on the RNA genomes of some *Flaviviridae* family members including HCV, ZIKV, dengue virus, yellow fever virus, and West Nile virus (Gokhale et al., 2016; Lichinchi et al., 2016b). Further studies found that during HCV infection, m^6^A modification negatively regulates virus replication. These findings raised the question of why these viruses would retain m^6^A if it negatively impacts their life cycles. One possible explanation is that the m^6^A modifications may facilitate viral escape from host antiviral immune responses. This speculation is supported by other studies using several *in vitro* synthesized m^6^A RNAs, which suppress recognition by host pattern recognition receptors, TLR3, TLR7, TLR8, and RIG-1 (Kariko et al., 2005; Durbin et al., 2016).

In investigating the potential involvement of reader proteins in flavivirus replication, DF1–3 were silenced to identify whether altered reader protein expression affects m^6^A-mediated negative regulation of ZIKV and HCV RNA. In both viruses, knockdown of DF1-3 increased the levels of extracellular viral RNA (Lichinchi et al., 2016b). Furthermore, in ZIKV-infected cells, these observations were verified by DF1–3 overexpression, which reduced extracellular viral RNA levels. The studies also showed the discriminatory binding of YTH proteins to HCV and ZIKV RNA by immunoprecipitation. All these results revealed that the modulation of RNA levels in HCV and ZIKV is functionally linked to DF binding of m^6^A methylated viral RNA. Additionally, by site-directed mutagenesis of four potential m^6^A sites on the E1 gene, they found that E1-mutated HCV RNA was bound more efficiently by the HCV core protein, enhancing its packaging into nascent virions. These data demonstrated a specific mechanism by which the reader proteins facilitate m^6^A-mediated negative regulation of viral replication, likely through competition with core proteins to bind the region of the E1 gene to suppress packaging of viral RNA into infectious viral particles.

### Enterovirus

*Enterovirus* is the most-studied genus in the *Picornaviridae* family, which is composed of positive, ssRNA viruses that replicate in the cytoplasm. Poliovirus (PV) and enterovirus are the typical representatives of the *Enterovirus* genus. To date, although m^6^A modifications in poliovirus RNA have been reported (McIntyre et al., 2018), their specific modification sites on the genome have not been assessed. Recently, m^6^A modification of enterovirus 71 (EV71) RNA has been investigated. It was found that m^6^A sites distribute in genes encoding the VP1 capsid protein, the 3D RNA-dependent RNA polymerase, and the non-structural protein 2C (Hao et al., 2019). Mutations of the m^6^A sites in VP1 and 2C genes decreased EV71 replication, suggesting that m^6^A modifications positively regulate EV71 infection. In determining the role of m^6^A reader proteins in EV71 replication, knocking down reader proteins, including DF1–3 and DC1, with siRNA in AD cells enhanced viral replication. However, knockdown and overexpression of DF2 and DF3 in Vero cells resulted in decreased and increased viral replication, respectively. In addition, this study also found that EV71 infection upregulated DF1–3 and DC1 expression and led to the partial relocalization of DF1 and DF2 to the nucleus, whereas the nuclear reader DC1 relocated to the cytoplasm. Another study of PV and coxsackievirus found that three readers DF1–3 were all cleaved by viral protease 2A at a very early phase of infection. The authors further demonstrated that DF3 acts as a positive regulator of antiviral JAK/STAT signaling in response to positive ssRNA virus infection, enhancing type I interferon (T1IFN)-mediated gene regulation in infected cells. The authors proposed that EV 2A proteases cleave DF proteins to antagonize interferon-stimulated gene (ISG) induction in infected cells (Kastan et al., 2021). For the epitranscriptomic analysis of host m^6^A after EV infection, a recent study using clinical samples from children infected with EV identified 957 m^6^A-modified cellular genes. The different m^6^A methylations were increased in CDS regions but decreased in the 3′UTR and stop codon regions in the neurological symptom group. The authors further revealed the differences in related cellular functions and signaling pathways associated with m^6^A methylation patterns (Zhu et al., 2021).

### Coronavirus

The genome of coronaviruses consists of a positive-sense ssRNA molecule. Coronaviruses also produce subgenomic RNAs. The first identification of m^6^A in a member of the *Coronaviridae* family was conducted in the porcine epidemic diarrhea virus (PEDV) (Chen et al., 2020), shortly followed by SARS-CoV-2 (severe acute respiratory syndrome coronavirus-2) (Kim et al., 2020b). PEDV, a member of the alpha-coronavirus genus, causes high mortality associated with severe diarrhea and vomiting in piglets younger than 1 week of age. m^6^A-seq revealed that the PEDV genome contains seven m^6^A peaks, mostly located in ORF1b, which encodes non-structural proteins. Depletion of m^6^A reader DF1 or DF2 increases PEDV replication while FTO depletion decreases replication. The reader proteins, especially DF2, inhibit viral replication by reducing viral RNA stability. SARS-CoV-2 is a member of the beta-coronavirus genus (Wu et al., 2020), mainly causing acute respiratory distress syndrome (ARDS) and acute cardiac injury (Huang et al., 2020; Xu et al., 2020). A recent study using combined m^6^A-seq and miCLIP analyses identified eight m^6^A modifications on the SARS-CoV-2 genome at single-base resolution (Liu et al., 2021a), mainly located at the ORF1ab, ORF7a, N, and ORF10 regions (Li et al., 2021; Liu et al., 2021a). However, since these experiments were carried out using fragmented total RNA, the authors could not confirm whether these modifications are on the genome or subgenomic RNA or both. Of the three DF1–3 readers, only depletion of DF2 affected viral replication, which was increased compared with control cells. Therefore, as in PEDV, m^6^A modifications negatively regulate SARS-CoV-2 infection. However, Burgess and colleagues presented a contrasting result when utilizing human A549 lung carcinoma cells. Their findings demonstrated that the replication of SARS-CoV-2 was hindered upon the depletion of METTL3 or DF1 and DF3 (Burgess et al., 2021). Moreover, the utilization of STM2457, a novel and highly selective small-molecule inhibitor of METTL3, markedly decreased the production of infectious HCoV-OC43 virus by over 100-fold and SARS-CoV-2 virus by 300-fold. As such, STM2457 represents a promising therapeutic agent to specifically target the m^6^A pathway and curb the reproduction of coronavirus.

### Chikungunya virus

Chikungunya virus (CHIKV) is a member of the *Togaviridae* family. Its genome is a positive-sense ssRNA molecule (Weaver and Lecuit, 2015). The m^6^A modifications on CHIKV RNA were identified using a novel method called *viral cross-linking and solid-phase purification* (VIR–CLASP**)**, which captures interactions between the pre-replicated viral genome and cellular proteins (Kim et al., 2020a). VIR–CLASP revealed that DF2 and DF3 interact with the CHIKV RNA. Moreover, m^6^A modifications were found to be abundant on the 5′ end of CHIKV genomic RNA. In addition, the effect of m^6^A on CHIKV replication is subject to combinatorial regulation by DF proteins. Knockdown and overexpression studies demonstrated the different effects of the three DF readers on viral RNA transcription, particularly on different (plus and minus) strands. Therefore, the authors moved on to testing whether the early effects of DF proteins on CHIKV persist until the release of new viral particles. They showed that knockdown of DF1 and DF3 increased both extracellular viral RNA levels and mature virions, while knockdown of DF2 had the opposite effect. These data suggest that DF1 and DF3 restrict CHIKV replication while DF2 promotes CHIKV replication (Kim et al., 2020a). It is worth highlighting that CHIKV expresses the viral non-structural and structural proteins from its genomic and subgenomic viral RNAs, respectively. However, whether the m^6^A modifications in the genomic and subgenomic RNAs are differentially regulated and carry out distinct functions during the complex viral life cycle are still unknown.

### Respiratory syncytial virus

Respiratory syncytial virus (RSV) is a non‐segmented, negative‐sense ssRNA virus in the *Pneumoviridae* family (Afonso et al., 2016). This virus mainly causes upper and lower respiratory tract infections in infants and children (Collins and Graham, 2008). m^6^A-seq analysis found that RSV viral negative-sense genomic RNA (gRNA), complementary RNA (cRNA), and the multiple viral RNA transcripts were internally m^6^A-modified (Xue et al., 2019). The viral G transcript encoding the attachment glycoprotein present on the surface of the RSV virion was the most extensively modified of the 10 viral mRNA transcripts. m^6^A writers positively regulated RSV replication, while m^6^A erasers showed the opposite effect. Overexpression of DF1–3 readers significantly increased RSV protein expression, gRNA and mRNA synthesis, and viral particle formation. It appears that overexpression of DF1–3 enhanced the ability of the RSV polymerase to synthesize both replicate and transcript. It should also be noted that overexpression of DF1 protein in A549 cells did not significantly affect the growth or survival of the host cells. Synonymous mutations of conserved m^6^A motifs under the three m^6^A peaks in the G transcript showed reduced viral replication and pathogenesis *in vitro* and in the respiratory tracts of cotton rats. Since m^6^A function is mediated *via* the binding of reader proteins, the authors also verified the specific binding of DF1–3 with the RSV G mRNA (Xue et al., 2019). Together, these results demonstrate a positive role for m^6^A in RSV infection. These findings highlight the viral m^6^A machinery as a possible novel target for rational design of live attenuated vaccines.

### Human metapneumonium virus

Human metapneumonium virus **(**HMPV) is the first human member of the Metapneumovirus genus in the *Pneumovirinae* subfamily within the *Paramyxoviridae* family. It is an enveloped negative-sense ssRNA virus. The genome includes eight genes coding for nine different proteins (van den Hoogen et al., 2002). m^6^A-seq analysis found that both the viral genome and anti-genome were m^6^A-modified and the strongest m^6^A peaks were in the G gene. The m^6^A peak regions identified in both strands are largely overlapping, and HMPV-induced m^6^A modifications promote HMPV replication (Lu et al., 2020). Not only does promotion of HMPV replication occur due to the action of writer proteins but also the reader proteins play an important role. For example, transient overexpression of DF1, DF2, DF3, or DC1 increases the levels of viral G and N proteins, as well as the release of infectious virus, anti-genome, and N and G mRNAs in A549 cells. This proviral function was also observed in HeLa cells stably overexpressing DF1, DF2, or DF3 using recombinant GFP-expressing HMPV (Lu et al., 2020). Although HMPV infection has a minimal impact on the methylation of cellular mRNAs, it significantly upregulates genes that are involved in type I IFN signaling, such as retinoic acid-inducible gene-I (RIG-I). These *in vitro* data were further supported by studies using cotton rats (Lu et al., 2020), suggesting that HMPV may utilize m^6^A modification to mimic host RNA and avoid detection by the innate immune system.

### Vesicular stomatitis virus and other non-segmented negative-sense RNA viruses

Vesicular stomatitis virus (VSV) is a negative‐sense, non‐segmented (NNS) ssRNA virus in the *Rhabdoviridae* family. m^6^A-seq revealed that its anti-genome contains 18 m^6^A sites (Qiu et al., 2021). During VSV replication, the produced double-stranded (dsRNA) intermediate serves as a signal in activating RIG-I‐like receptors (RLRs) that recognize RNA intracellularly, triggering an antiviral mechanism. However, m^6^A readers can function as trans‐acting factors to compete with RLRs and inhibit RLRs from binding to m^6^A-modified RNAs (Lu et al., 2020). Another study found that among the three DF1–3 readers, only DF3 suppressed ISG expression under basal conditions by promoting translation of the transcription corepressor forkhead box protein O3 (FOXO3) (Zhang et al., 2019b). In this process, DF3 cooperated with two cofactors, PABP1 and eIF4G2, to promote FOXO3 translation by binding to the translation initiation region of FOXO3 mRNA. Moreover, DF3 KO mice had increased ISG levels and were resistant to several viral infections. These findings uncover the role of DF3 as a negative regulator of antiviral immunity. However, a recent study, using VSV and several other NNS RNA viruses including HMPV in the *Pneumoviridae* family and SeV (Sendai virus) and MeV (measles virus) in the *Paramyxovirida*e family, found that m^6^A on the genomes and anti-genomes of several families of NNS RNA viruses enabled viral RNA to escape recognition by RIG-I. In addition, the m^6^A reader DF2 is essential for suppression of T1IFN responses (Lu et al., 2021). These data suggest that the three families of NNS RNA viruses have evolved a common mechanism, which is to mask their genome and anti-genome with m^6^A, to mimic the host RNA and evade host innate immunity that is dependent on RNA sensor RIG-I.

### Rotavirus

Rotavirus (RV), a member of the family *Reoviridae*, is a non-enveloped virus with 11 segments of dsRNA. Children under the age of five are at high risk of rotavirus infection, which causes severe diarrhea, dehydration, and death (Crawford et al., 2017). RV infection induces global m^6^A modifications on host mRNA transcripts by down-regulating the m^6^A eraser ALKBH5. During RV infection, m^6^A modification of small bowel intestinal epithelial cells (IECs) has a negative effect on IFN response. Specifically, mice lacking the m^6^A writer enzymes METTL3 in IECs (Mettl3ΔIEC) showed increased expression of IFNs and ISGs and thus were resistant to RV infection (Wang et al., 2022). This study further found IRF7 to be one of the primary m^6^A modification targets during virus infection. In the absence of METTL3, IECs showed increased Irf7 mRNA stability and enhanced type I and III IFN expression. Deficiency in IRF7 attenuated the elevated expression of IFNs and ISGs and restored susceptibility to RV infection on Mettl3ΔIEC mice. However, in this m^6^A-IRF7-IFN signal cascade, the functional roles of reader proteins have not been elucidated.

### Influenza virus

Influenza A virus (IAV) is a member of the *Orthomyxoviridae* family, which is composed of negative, segmented, ssRNA viruses that replicate in the nucleus. IAV was the first virus found to express mRNA with internal m^6^A residues; the highest number of m^6^A modifications was detected on hemagglutinin (HA) mRNA segments encoding the viral envelop protein (Krug et al., 1976; Narayan et al., 1987). Recently, Courtney et al. reported that m^6^A modification of IAV RNA positively regulates viral gene expression and virion production as mutating m^6^A sites by CRISPR/Cas9 or inhibition of METTL3 with inhibitor 3-deazaadenosine (DAA) reduced the levels of both viral mRNA and proteins (Courtney et al., 2017). By using the PAR-CLIP method with three reader proteins DF1–3 as well as the PA-m^6^A-seq technique to map the m^6^A residues in IAV-infected cells, the authors found that m^6^A residues were present on multiple locations of the viral mRNA encoding structural proteins and at lower levels on the mRNA-encoding RNA polymerase subunits. A recent large-scale comparative m^6^A analysis using 70,030 complete HA sequences was conducted to investigate the conservation patterns of the DRACH motifs (Bayoumi and Munir, 2021). This bioinformatic analysis revealed the highest degree of DRACH conservation among all H1 sequences that clustered largely in the middle and in proximity of the 3′ end with at least four DRACH motifs conserved in all mRNA sequences. Interestingly, the total number and the conserved DRACH motifs in the viral RNA were found to be much lower than those observed in the mRNA. In their functional study of the readers in IAV infection, Courtney et al. reported that although DF1 and DF3 did not show an effect on viral replication, DF2 overexpression significantly promoted IAV replication and viral particle production. Taken together, this study provides evidence to suggest that m^6^A modifications in IAV RNA positively regulate viral infection and pathogenesis. Although the underlying mechanism is unclear thus far, this positive regulation is not likely through the downregulation of host antiviral innate immune response proteins, including RIG-1, MGA5, and interferon-β (Courtney et al., 2017).

### HIV and other retroviruses

The members of the *Retroviridae* family are positive ssRNA viruses that replicate in the nucleus. m^6^As have been identified in retroviruses, including human immunodeficiency virus-1 (HIV-1), Rous sarcoma virus, and feline leukemia virus (Gokhale and Horner, 2017; Riquelme-Barrios et al., 2018). Several years ago, three independent groups reported the mapping of m^6^A residues on viral RNA and further characterization of the involvement of m^6^A in HIV replication; however, their findings were not consistent (Kennedy et al., 2016; Lichinchi et al., 2016a; Tirumuru et al., 2016; Lu et al., 2018; Riquelme-Barrios et al., 2018). For example, all three reports showed an enrichment of m^6^A residues at the 3′ and/or 5′ UTR of HIV‐genomic RNA, but two of the studies discovered additional m^6^A residues along the viral genome (Lichinchi et al., 2016a; Tirumuru et al., 2016). This discrepancy may be due to the use of different cell lines, viral strains, and/or mapping techniques.

In functional studies of m^6^A, a group reported that overexpression of readers DF1–3 inhibited viral replication by blocking viral reverse transcription and promoting degradation of incoming viral RNA (Tirumuru et al., 2016). This finding was further solidified by their studies using virus-producing cells, in which they found that three DF1–3 readers preferentially bound to the m^6^A-modified leader sequence of viral genome RNA compared with the unmodified RNA counterpart (Lu et al., 2018). This finding on the negative role of reader proteins in HIV-1 replication is opposite to the results of Kennedy’s group. Kennedy et al. found that overexpression of DF1–3 in 293T cells increased gene expression of viral *gag*, *ref*, *tat*, and *rev* mRNAs and Gap and Nef proteins. In further studies focusing on DF2 in CEM-SS cells, they found that overexpression and knockdown of DF2 could increase and decrease HIV-1 replication, respectively (Kennedy et al., 2016). This inconsistency may be due to the cell lines or experimental conditions applied.

Recently, another study found that both DC1 and DF2 could bind to multiple distinct and overlapping sites on the HIV-1 RNA, with DC1 recruitment serving to regulate the alternative splicing of HIV-1 RNAs, while DF2 binding to m^6^A residues present on cellular mRNAs resulted in their destabilization, as previously reported, and DF2 binding to m^6^A sites on HIV-1 transcripts resulted in a marked increase in the stability of these viral RNAs. Thus, DF2 binding can exert diametrically opposite effects on RNA stability, depending on the RNA sequence context (Tsai et al., 2021). The finding of the role of DC1 in promoting viral infectivity was supported by another study (N'Da Konan et al., 2022). For reader DF3, it was reported that DF3 was incorporated into HIV particles in a nucleocapsid-dependent manner and reduced viral infectivity in the next cycle of infection. Interestingly, the authors found that HIV protease cleaves the virion-incorporated full-length DF3 protein, a process that can be blocked by FDA-approved HIV protease inhibitors (Jurczyszak et al., 2020).

In addition to the reader proteins, Lichinchi et al. also characterized the function of two m^6^A sites within the Rev response element (RRE) of HIV. The RRE is an important structural and functional RNA element within the HIV-1 *env* gene coding for an HIV-1 envelope protein. Lichinchi et al. found that m^6^A modification on viral RNA positively affected the interaction between HIV Rev protein and RRE RNA. This promoted the formation of Rev-RRE complexes and the nuclear export of viral transcripts, and hence viral replication (Lichinchi et al., 2016a). Although the mechanism by which m^6^As promote the binding of Rev to the RRE in cells is not clear, it is possible that methylation can alter the RNA structure of RRE (Liu et al., 2015; Liu et al., 2017), which facilitates Rev protein binding. An alternative explanation is that Rev is a viral m^6^A reader with higher affinity for methylated viral transcripts. Taken together, all these data indicate that m^6^A and its machinery have multiple roles in regulating HIV-1 infection depending on the stage of the viral life cycle, the position of m^6^A in the HIV-1 RNA, and the binding of different readers.

### Endogenous retroviruses

Endogenous retroviruses (ERVs) are abundant and heterogeneous groups of integrated retroviral sequences that affect genome regulation and cell physiology throughout their RNA-centered life cycle (Johnson, 2019). Using a genome-scale CRISPR knockout screen in mouse embryonic stem (ES) cells, Chelmicki et al. identified m^6^A RNA methylation as a way to restrict ERVs and that methylation of ERV mRNAs is catalyzed by the complex of METTL3/14 proteins. They also found that depletion of this writer complex, along with accessory subunits WTAP and ZC3H13, led to increased mRNA abundance of intracisternal A-particles (IAPs) and related ERVK elements. The authors further revealed that the m^6^A-dependent repression was proportional to the m^6^A content in the 5′UTR: the more m^6^A sites an IAPEz copy contains, the more upregulated it is in m^6^A-knockout cells. In addition, using controlled auxin-dependent degradation of this writer complex, they showed that IAP mRNA and protein abundance is dynamically and inversely correlated with m^6^A catalysis. Furthermore, using two mutant ES cell lines that exhibited loss of all three DF1–3 proteins, they found that depletion of DFs increased IAP mRNA levels, which supports the hypothesis that the m^6^A methylation pathway regulates IAP mRNAs in a posttranscriptional and DF-dependent manner.(Chelmicki et al., 2021) These results indicate that RNA methylation provides a protective effect in maintaining cellular integrity by clearing reactive ERV-derived RNA species, which may be especially important when transcriptional silencing is less stringent.

## m^6^A and its reader proteins in DNA viral replication

### Simian virus 40

Simian virus 40 (SV40) is a small, double-stranded DNA (dsDNA) virus in the *Polyomaviridae* family. This virus replicates in the nucleus and often causes human tumors (Vilchez et al., 2003). The m^6^A modifications of SV40 transcripts were found over 40 years ago (Canaani et al., 1979; Finkel and Groner, 1983). However, the precise location of the m^6^As on SV40 viral mRNA and their functional significance in SV40 replication were unknown until recently. Tsai et al. conducted studies modifying the expression levels of the m^6^A machinery to identify any associated changes in viral replication (Tsai et al., 2018) and found that overexpression of DF2 elevated the expression of both the early large T antigen protein and the late structural protein VP1, leading to enhanced viral particle formation. However, a much less profound effect on viral replication was observed upon overexpression of DF3. Moreover, deletion of both DF2 and METTL3 reversed the effects observed following DF2 overexpression. These data indicate that m^6^A modification in SV40 RNA plays a positive role in the viral life cycle.

To further understand the underlying mechanism of these observations, the authors mapped the sites of m^6^As and found 13 peaks within the SV40 genome including 2 in the early transcript and 11 in the late transcript, most of which were present in the open reading frame (ORF) of structural protein VP1. They further conducted mutations of m^6^A on VP1 late transcript and found that mutations did not affect splicing but significantly decreased the VP1 protein levels despite the lack of significant changes in mRNA abundance. Nevertheless, comparing both cytosolic and nuclear fractions, a significant decrease in the VP1 transcripts was found in the cytosolic fraction, suggesting that m^6^A-mediated positive regulation of SV40 replication is due to both enhanced nuclear export and increased translation of viral late transcripts. In support of these findings, the addition of a global inhibitor of methylation, DAA, profoundly decreased SV40 late protein production (Tsai et al., 2018).

### Hepatitis B virus

Hepatitis B virus (HBV) is a dsDNA virus belonging to the *Hepadnaviridae* family, which replicates in the nucleus through the reverse transcription of an RNA intermediate known as pregenomic RNA (pgRNA). A recent study found that m^6^A modification occurs in both HBV pgRNA and mRNA (Imam et al., 2018). In studying the effect of m^6^A methylation on the HBV life cycle, the authors found that knockdown of METTL3/14 increased the expression of the viral proteins, while the opposite result was obtained in cells lacking FTO or ALKBH5 expression. These results suggest that m^6^A negatively regulates the expression of HBV proteins. Interestingly, an increase in the expression of pgRNA was also observed for DF2 and DF3 knockdowns, suggesting that the decrease in HBV protein levels is attributed to the reduction of RNA abundance rather than decreased translation. Further m^6^A-seq analysis identified an m^6^A peak at the conserved motif situated within the epsilon stem loop, which is located in the 3′ terminus of all HBV mRNAs and both the 5′ and 3′ termini of pgRNA. Mutational analysis of the m^6^A site in the 5′ epsilon stem loop of pgRNA revealed that m^6^A at this site was required for efficient reverse transcription of pgRNA, while m^6^A methylation of 3′ epsilon stem loop resulted in destabilization of all HBV transcripts, suggesting that m^6^A has a dual functional role in the HBV life cycle.

The RIG-I signaling pathway in IFN induction is one of the host defense systems to eliminate viral infections. A recent study demonstrated that cellular m^6^A machinery regulates the RIG-I signaling pathway activated by virus infection (Kim et al., 2020c). Specifically, the authors revealed that DF2 inhibits RIG-I recognition of viral RNAs *via* interacting with m^6^A-modified viral RNAs, leading to a disrupted RIG-I-mediated immune response. However, another study revealed that DF2 could form a complex with IFNα-induced ISG20, a 3′–5′ exonuclease enzyme and selectively target the m^6^A-modified HBV transcript to perform RNA degradation (Imam et al., 2020). The functional roles of other readers were also investigated. It was reported that DC1 as well as FMRP recognized m^6^A-methylated HBV transcripts and facilitated their transport to the cytoplasm. In cells depleted with DC1 or FMRP, viral transcripts accumulated in the nucleus to affect the viral life cycle (Kim et al., 2021).

### Adenovirus

Adenovirus, a member of the *Adenoviridae* family, is a nuclear replicating dsDNA virus. While the m^6^A modification in viral transcripts was discovered in 1970s (Sommer et al., 1976; Chen-Kiang et al., 1979), the location of this methylation was unknown, largely due to the fact that the complex adenovirus transcriptome includes overlapping spliced units that impede accurate m^6^A mapping. A recent study using a combination of meRIP-seq and direct RNA long-read sequencing to profile the m^6^As within the transcriptome yielded both nucleotide- and transcript-resolved m^6^A detection (Price et al., 2020). They also found that adenovirus infection did not alter the expression of m^6^A machinery, such as writers (METTL3, METTL14, and WTAP) and readers (DC1, DF1, and DF2). These host proteins remained unchanged but were concentrated at sites of nascent viral RNA synthesis. Although both early and late viral transcripts contain m^6^As, depletion of METTL3 caused significantly more reduction of gene expression in viral late RNAs than in early RNAs. This late gene-biased effect was primarily mediated by decreased RNA splicing efficiency in the absence of METTL3 and could be extended to all of the multiply spliced adenovirus late RNAs (Price et al., 2020). Overall, these results suggest positive regulation of adenovirus by m^6^A modification and highlight the role of m^6^A in regulating the splicing and expression of a viral pathogen.

### Herpesviruses

Members of the *Herpesviridae* family are large, dsDNA viruses. They replicate in the nucleus, causing distinct lytic and latent infections, as well as oncogenesis. While decades ago, herpes simplex virus type 1 (HSV-1) was found to contain m^6^A in its mRNA (Moss et al., 1977), m^6^A has now been mapped and functionally characterized in cells infected with human cytomegalovirus (HCMV) (Rubio et al., 2018; Winkler et al., 2019), Epstein–Barr virus (EBV) (Zheng et al., 2021), and Kaposi’s sarcoma-associated herpesvirus (KSHV) (Ye et al., 2017; Hesser et al., 2018; Tan et al., 2018).

A recent report revealed that the HSV-1 genome transcript contains 12 m^6^A peaks located mainly in the upstream, overlapping start, and inside the transcript region covering multiple viral transcripts. Viral infection enhances the expression of METTL3/14 and DF1–3 at an early stage and decreases their expression at the late stage; however, the expression of ALKBH5 and FTO is consistently suppressed during infection (Feng et al., 2022). Furthermore, inhibiting m^6^A modification by DAA or silencing writer and reader genes by siRNAs significantly decreases viral replication, whereas depleting the erasers or ectopically expressing METTL3 promotes viral replication. These data suggest that m^6^A modification benefits HSV-1 replication. Another study found that HSV-1 infection caused redistribution of nuclear m^6^A machinery including reader DC1. siRNA silencing of m^6^A methyltransferase reduced viral gene expression initially but less so as the infection advanced. The authors further found that this redistribution was accompanied by a wide-scale reduction in m^6^A addition and other RNA modifications on both host and viral mRNAs. Thus, they indicated that the m^6^A pathway is important for HSV-1 gene expression at the beginning of the replication cycle, becoming dispensable later (Srinivas et al., 2021).

HCMV has the largest genome among members of the *Herpesviridae* family and is best known for its propensity to cause disease in immunocompromised patients, especially transplant recipients, patients with advanced AIDS, and congenitally infected newborns (Boeckh and Geballe, 2011). Two recent studies in HCMV focused on how m^6^A impacted antiviral innate immunity *via* regulation of IFN production (Rubio et al., 2018; Winkler et al., 2019). One reported that in HCMV-infected or dsDNA-treated cells, deletion of METTL3 or DF2 led to an increase in the induction of ISGs. Consequently, viral propagation was suppressed in an interferon-signaling-dependent manner. Another study observed similar results by altering the expression levels of METTL14 or ALKBH5 demethylase (Rubio et al., 2018). Significantly, both studies found that mRNA of IFNβ was m^6^A-modified and stabilized following repression of METTL3, METTL14, or DF2. In addition, the former study showed that m^6^A-mediated regulation of interferon genes is conserved in mice. Mice lacking DF3 exhibited enhanced *Ifna* and *Ifnβ* induction following viral infection (Winkler et al., 2019). Together, these findings suggest that m^6^A serves as a negative regulator of the T1IFN response by dictating the fast turnover of IFNα and IFNβ mRNAs and consequently facilitating viral propagation.

EBV is a ubiquitous oncogenic virus that induces many types of cancers. EBV infection has three phases: the prelatent, latent, and lytic phases (Hammerschmidt, 2015). During infection, METTL14 expression is induced, and knockdown of METTL14 leads to decreased latent EBV gene expression. METTL14 is also significantly induced in EBV-positive tumors, promoting growth of EBV-transformed cells and tumors in xenograft animal models. Mechanistically, the virus-encoded latent oncoprotein EBNA3C activates transcription of METTL14 and promotes its stability by directly interacting with METTL14. This study demonstrated that EBV hijacks METTL14 to drive EBV-mediated tumorigenesis (Lang et al., 2019). Another study, examining the viral and cellular m^6^A epitranscriptomes, found that in the pre‐latent phase, EBV EBNA2 and BHRF1 were highly m^6^A‐modified upon EBV infection (Zheng et al., 2021) and that knockdown of METTL3 decreased EBNA2 expression. Further, Xia et al. demonstrated that EBV transcripts exhibited differential m^6^A modifications in human nasopharyngeal carcinoma biopsies, patient-derived xenograft tissues, and cells at different EBV infection stages (Xia et al., 2021). m^6^A-modified EBV transcripts can be recognized and destabilized by the DF1 reader, which leads to m^6^A-dependent suppression of EBV infection and replication. Mechanistically, DF1 accelerates viral RNA decapping and mediates RNA decay by recruiting RNA degradation complexes, including ZAP, DDX17, and DCP2, thereby posttranscriptionally downregulating the expression of EBV genes.

KSHV is the most studied virus in the *Herpesviridae* family thus far. Several groups have mapped the m^6^A sites in different transcripts of the virus during KSHV infection. These include KSHV *ORF50/RTA* mRNA, which encodes the replication and transcription activator (RTA) required for the reactivation of latent KSHV (Baquero-Perez and Whitehouse, 2015). While three of these early studies showed that m^6^A regulates RTA, they presented conflicting results for how m^6^A and its machinery control the *ORF50/RTA* RNA levels and/or expression, which might be due to the different cell types used. In B cells, Ye et al. identified several m^6^A sites crucial for RTA pre-mRNA splicing that were bound by m^6^A reader DC1 and its associated splicing factors, SRSF3 and SRSF10. They also found that the lytic switch protein RTA itself strongly induced m^6^A modification and enhanced its own pre-mRNA splicing (Ye et al., 2017). However, in the same cells, Hesser et al. found a negative effect of m^6^A on *ORF50/RTA* and KSHV lytic reactivation (Hesser et al., 2018). In epithelial cells, Tan et al. found that DF2 depletion increased the stability of many viral transcripts including *ORF50/RTA* during reactivation, which might or might not change its expression, indicating a potential role for m^6^A in KSHV lytic reactivation (Tan et al., 2018). Conversely, Hesser et al. showed that METTL3 and DF2 depletion suppressed KSHV *ORF50/RTA* expression, lytic reactivation, and viral particle release, suggesting that m^6^A promotes KSHV lytic reactivation (Hesser et al., 2018).

The m^6^A nuclear reader DC2 was also studied in KSHV-infected cells for its function in stabilizing RNAs, since many cellular mRNAs are degraded by KSHV endoribonuclease SOX (Macveigh-Fierro et al., 2022). This study found that the IL6 mRNA was m^6^A modified in its 3′UTR during KSHV lytic infection and that removal of this m^6^A restored susceptibility to SOX-mediated degradation. They further showed that DC2 bound to the IL6 SOX resistance element in an m^6^A-dependent manner and that downregulation of DC2 was sufficient to abrogate resistance to SOX. These results indicate that the m^6^A pathway is pivotal in the regulation of gene expression during KSHV infection, highlighting the viral-host battle for control of RNA stability. In addition to the two m^6^A readers mentioned above, a recent study reported a new m^6^A reader, SND1 (Staphylococcal nuclease domain-containing protein 1), for the lytic reactivation of KSHV through binding to ORF50/RTA. The authors found that m^6^A modification of the ORF50/RTA RNA was critical for SND1 binding, which in turn stabilized the ORF50 transcript. The SND1 binding was hairpin structure-dependent and preferable to the unspliced full length of ORF50/RTA. Deletion of SND1 led to inhibition of KSHV early gene expression, suggesting that SND1 is essential for KSHV lytic replication (Baquero-Perez et al., 2019).

### Bombyx mori nucleopolyhedrovirus

BmNPV, a representative member of the *Baculoviridae* family, specifically infects the silkworm (Bombyx mori) and causes serious economic losses to the silkworm industry (Zhang et al., 2019a). BmNPV is an enveloped circular DNA virus that replicates in the nucleus. MeRIP sequence analyses found that m^6^A modifications are widespread in BmNPV transcripts in viral infected cells and predominantly appear in the coding sequences (CDS) and the 3′-end of the CDS. Among the viral genes related to replication and proliferation, *ie-1* mRNA was found to have a higher m^6^A level than other viral genes. The m^6^A sites in the *ie-1* mRNA may be negatively related to the protein expression of this mRNA. Cells overexpressing DF3 inhibited viral replication in a dose-dependent manner, and an opposite effect was found when silencing DF3 with siRNAs (Zhang et al., 2022).

## M^6^A and its readers in virus-induced innate and adaptive immunity

Recent studies on viral m^6^A modifications have shifted from analyzing the m^6^A landscape and its effect on viral replication to investigating its regulatory mechanisms. In this regard, the T1IFN immune response is a major focus as it activates intracellular antimicrobial programs and influences the development of innate and adaptive immune responses (Shulman and Stern-Ginossar, 2020). For detailed information, readers can refer to recent reviews (Lou et al., 2021; Tang et al., 2021). The innate immune response is the first line of host antiviral response and can discriminate cellular (self) from viral (non-self) RNAs using pathogen recognition receptors (PRR). PRRs include toll-like receptor (TLR) 3, 7, and 9 in endosomes and the RIG-I like receptors (RLR) in the cytosol, of which the most important are RIG-I and the melanoma differentiation-associated protein 5 (MDA5) (Wu et al., 2014; Chow et al., 2018). Upon recognition by these RNA sensors, intracellular signaling cascades are activated, triggering the expression and secretion of IFNα and IFNβ that are recognized by the IFN receptor, activating the downstream JAK-STAT pathway. This pathway leads to transcription of a number of ISGs that establish an antiviral response (Ivashkiv and Donlin, 2014). Viruses have evolved to escape or inhibit the T1IFN response using multiple strategies, which have been very briefly mentioned above in each virus group based on availability of data; here, we would like to make a summary of the three major mechanisms by which m^6^A modifications modulate host immune responses.

## Direct m^6^A modification of viral transcripts to escape host immune recognition

RNA structure and posttranscriptional modifications are important molecular markers for the PRR to discriminate self from non-self RNAs. The primary ligand of RIG-I is a 5′ triphosphated or diphosphated ssRNA (Hornung et al., 2006; Pichlmair et al., 2006). Both MDA5 and RIG-I can recognize dsRNAs; MDA5 preferentially detects long dsRNA, while RIG-I detects short dsRNA (Kato et al., 2008; Schlee et al., 2009; Runge et al., 2014). Cellular mRNAs contain a unique 5′cap structure typically methylated at the guanine N7 and ribose-2′-O-position (Furuichi et al., 1977; Shuman, 2002), and this structure is not detected by RIG-I and MDA5 (Hyde and Diamond, 2015). mRNAs lacking the ribose-2′-O-methylation are recognized as the foreign RNA. Thus, certain viruses use either their own methyltransferases (e.g., WNV and VSV) (Hyde and Diamond, 2015; Johnson et al., 2018) or cellular methyltransferase FTSJ3 (e.g., HIV) to add ribose-2′-O-methylation to the RNA cap or internal regions (Ringeard et al., 2019). An additional methylation strategy is the utilization of cellular m^6^A methyltransferases to add m^6^A to viral RNA transcripts. For example, HMPV RNA uses cellular m^6^A machinery to add m^6^A and evade T1IFN detection. This notion is supported by data showing that m^6^A-deficient HMPV RNA induces a higher expression of RIG-I, binds efficiently to RIG-I, and facilitates the conformational change of RIG-I required to stimulate the IFN induction pathway. This finding was also verified *in vivo* using cotton rats infected with HMPV mutant lacking multiple m^6^A sites (Lu et al., 2020). Moreover, depletion of METTL3 or DF2 induces the mRNA expression of IFNβ and downstream ISGs in cells infected with HCMV, MCMV, IVA, adenovirus, or VSV (Winkler et al., 2019). Another example is RNA m^6^A modification of HIV, which suppresses the expression of antiviral cytokine T1IFN in differentiated human monocytic cells and primary monocyte-derived macrophages. Mechanistically, m^6^A of HIV-1 RNA escapes RIG-I-mediated RNA sensing and activation of the transcription factors IRF3 and IRF7 that drive IFN-I gene expression (Chen et al., 2021). Similarly, in HCV or HBV infection, depletion of METTL3/14 leads to an increase in viral RNA recognition by RIG-I, thus activating IFN production. However, overexpression of METTL3/14 reverses this outcome. The m^6^A modification of viral RNA renders RIG-I less effective, whereas mutation of the m^6^A consensus motif enhances RIG-I sensing activity. Importantly, DF2 and DF3 inhibit RIG-I-transduced signaling activated by viral RNAs by competitive binding to the m^6^A site to inhibit RIG-I recognition (Kim et al., 2020c). Consistently, METTL14 depletion reduces viral reproduction and stimulates HCMV- or dsDNA-induced IFNβ1 mRNA production while ALKBH5 depletion had the opposite effect (Rubio et al., 2018). This m^6^A-regulated innate immune response was also studied in several NNS RNA viruses including VSV, RSV, and HMPV (Lu et al., 2021; McFadden et al., 2021; Qiu et al., 2021). Qiu et al. found that m^6^A addition to VSV RNA decreased viral dsRNA formation, thereby reducing virus-sensing efficiency by RIG-I and MDA5 and dampening antiviral immune signaling. However, ablation of METTL3 enhanced T1IFN signaling and accelerated VSV clearance (Qiu et al., 2021). Similar results reported by Lu et al. showed that NNS RNA viruses utilized m^6^A as a common strategy to evade host immunity, which was supported by the fact that m^6^A-deficient viral RNA triggered a significantly higher level of T1IFN compared to m^6^A-sufficient viral RNA in a RIG-I-dependent manner. In addition, reader DF2 is essential for suppression of the T1IFN pathway (Lu et al., 2021).

## m^6^A modification of cellular mRNAs encoding the type I IFN signaling molecules

Among the host molecules involved in the T1IFN signaling, IFNβ mRNA is one of the major targets. An early study found that IFNβ1 mRNA was m^6^A modified within both the coding sequence and the 3′UTR in HCMV-infected or dsDNA-treated cells, and these data supported the hypothesis that m^6^A machinery controls IFNβ production (Rubio et al., 2018). Later, it was found that loss of m^6^As within IFNβ1 mRNA due to METTL3 or DF2 depletion leads to the stabilization of IFNβ1 mRNA and a stronger antiviral response during HCMV infection, suggesting that m^6^As may serve as a negative regulator of the antiviral IFN response (Winkler et al., 2019). Other antiviral adaptor molecules, such as TRAF3, TRAF6, and MAVS in the TIIFN signaling pathway, are also the targets of m^6^A modification. During innate immune stimulation, the mRNAs encoding these molecules lose their m^6^As, catalyzed by the eraser ALKBH5 recruited to these mRNAs by RNA helicase DDX46. The loss of m^6^As reduces their nuclear export and translation and thus inhibits IFN production (Zheng et al., 2017). Similarly, another DDX family member, DDX3, has been found to interact with ALKBH5 during the immune response, suggesting that this group of proteins may regulate viral infection by altering the abundance and stability of these molecules (Shah et al., 2017). On the other hand, the study also showed that METTL3 promotes the splicing of the TLR signaling adaptor MYD88 and the induction of several cytokines in response to lipopolysaccharide stimulation, which indicates that m^6^A can also positively regulate innate immunity (Feng et al., 2018). The activation of IFN signaling induces the expression of hundreds of ISG, which is also likely regulated by m^6^A and its machinery. For example, DF3 was reported to negatively regulate IFN signaling and subsequent ISG expression through promotion of translation of a transcriptional repressor of ISGs named FOXO3 (Zhang et al., 2019b). Consequently, knockdown of DF3 in macrophages inhibited infection of several viruses including VSV, EMCV, and HSV-1. However, McFadden et al. showed a positive effect of m^6^A on antiviral activity of T1IFN signaling. They found that IFITM1, an antiviral ISG, was upregulated at the translational level by m^6^A and the writer proteins METTL3/14. Furthermore, reader DF1 was found to increase the expression of IFITM1 in an m^6^A-binding-dependent manner (McFadden et al., 2021). Another m^6^A reader, hnRNPA2B1, can recognize DNA virus HSV-1 in the nucleus. Upon recognition and further dimerization and demethylation, hnRNPA2B1 translocates to the cytoplasm where it activates the T1IFN signaling pathway. Additionally, hnRNPA2B1 promotes m^6^A modification, nucleocytoplasmic trafficking, and translation of cGAS, IFI16, and STING mRNAs to fully ensure the IFNα/β production (Zhang et al., 2020). Other m^6^A readers, such as FMR1 and IGFBP3, also selectively recognize m^6^A in viral or cellular mRNAs and modulate their function during the antiviral response (Arguello et al., 2017; Edupuganti et al., 2017).

## m^6^A modification of cellular mRNAs encoding proteins regulating adaptive immunity

The adaptive immune response is another arm of the immune system that specializes in the clearance of specific pathogens. It is mediated by the activation of antigen-specific T and B lymphocytes, ultimately establishing long-lasting immune memory against the given antigen. Accumulated evidence reveals that m^6^A exerts a vital effect on adaptive immunity through targeting certain genes regulating immune cell homeostasis. For example, early studies using conditional METTL3 knockout mice reported a novel mechanism whereby m^6^A functions *in vivo* to control T-cell differentiation and proliferation by inducible degradation of mRNAs encoding suppressor of cytokine signal (SOCS) and consequently relieves blockage of IL7 signaling and T-cell proliferation (Li et al., 2017b). This notion is based on the fact that m^6^A modification targets the IL-7/STAT5/SOCS signaling pathway. The IL-7/STAT5 signal axis is critical for maintaining T-cell differentiation and proliferation. SOCS-1 and -3 could act as mediators binding to the IL-7 receptor, thus preventing STAT5 activation and downstream signaling to regulate T-cell homeostasis. Thus, m^6^A-mediated degradation of SOCS mRNA promotes T-cell proliferation and differentiation. The same research group later found that m^6^A was also essential for T regulatory (Treg) cell generation and suppression. Similarly, the m^6^A-mediated SOCS mRNA degradation activated the IL2/STAT5 signaling pathway to sustain the suppressive function and stability of Treg cells (Tong et al., 2018).

In addition to the positive effect of m^6^A on T-cell homeostasis, m^6^A RNA modification might exert an inhibitory effect on follicular help T (Tfh) cell differentiation. A recent study revealed that E3 ligase VHL promoted Tfh development and function at the early phase upon viral infection *via* suppression of hypoxia-inducible factor 1a (HTF-a1)-mediated glycolysis (Zhu et al., 2019). Mechanistically, VHL deficiency results in activation of the HIF-a1-GAPDH glycolytic pathway and consequently reduction in ICOS (inducible costimulatory) expression by enhancing m^6^A modification on ICOS mRNA, ultimately leading to attenuated Tfh cell differentiation.

Furthermore, m^6^A modification also exerts essential effects on early B-cell development. Zheng et al. demonstrated that loss of writer METTL14 blocked two key transitions in B-cell development: (i) METTL14-mediated m^6^A modification facilitates IL-7-induced pro-B cell proliferation *via* its reader DF2; (ii) the large-pre-B-to-small-pre-B transition is independent of reader DF1/2. Actually, this transition is largely dependent on the METTL14-mediated proper transcriptional activation of several transcription factors (Zheng et al., 2020). Another study showed that in diffuse large B-cell lymphoma (DLBCL), METTL3 expression and m^6^A level were increased in both tissue and cell lines. Upregulated METTL3 promoted DLBCL cell proliferation by increasing the mRNA level of pigment epithelium-derived factor through m^6^A modification (Cheng et al., 2020). Similarly, WTAP was also found to be involved in the induction of DLBCL cell proliferation by enhancing m^6^A modification of HK2 mRNA, leading to HK2 upregulation. However, in this circumstance, WTAP was upregulated by piRNA-30743 and thus DLBCL tumorigenesis was through a piRNA/WTAP/HK2-m^6^A axis(Han et al., 2021). Collectively, these studies indicate that m^6^A modification plays an important role in early B-cell development.

## Conclusions and future perspectives

With the advances in high-throughput MeRIP-seq techniques, in recent years, our understanding of the m^6^A landscape and its roles in regulating viral replication has greatly advanced. Study findings indicate that m^6^A may be proviral or antiviral depending on the viral species and which step is targeted during the viral life cycle (Zaccara et al., 2019; Baquero-Perez et al., 2021). To date, many viruses have been found to be m^6^A-modified in their RNA transcripts and therefore regulated by m^6^A machinery. Deletion or overexpression of the m^6^A writer, eraser, or reader proteins affects diverse facets of viral replication that are often mediated by RNA-binding protein interactions. This implies that the reader proteins of the m^6^A-containing transcripts play an executive role in this regulatory process. In this regard, the reader proteins may bind not only to the m^6^A sites of viral RNA but also to those of host transcripts. Thus, m^6^A-mediated regulation of viral replication is also tissue- or cell-type dependent. In brief, viral replication is regulated by m^6^A and their machinery, particularly the various readers, in a way that is more complex than was expected. Future experiments studying m^6^A in viral infection should focus on the specific modification sites of viral transcripts, their effects on the temporal dynamics of methylation, and the functions of newly identified readers.

Previous studies largely focused on identifying the location of m^6^A clusters in viral RNAs. With the development of new techniques to increase the resolution of mapping at the single-nucleotide level, it is possible to study the role of specific m^6^A sites on viral transcripts in viral propagation by site-directed mutagenesis. In addition, since m^6^A modification is a dynamic and reversible process, it is very important to determine whether dynamic changes in m^6^A modifications correspond to altered viral replication efficiency at specific time points during infection. Furthermore, we can identify which sites are critical for these changes. As reader-binding relies on the m^6^A site and its surrounding conserved motif, it is very interesting to determine whether reader binding is also a dynamic process; if so, further determination of which m^6^A methylations are responsible for this dynamic change should be conducted. Additionally, it is known that m^6^A methylation affects RNA structures and that certain RNA-binding proteins require specific structures for maximum binding capacity. By identifying m^6^A sites at the single-nucleotide resolution, it is possible to computationally predict which modifications can significantly alter RNA secondary structure. These predictions can then be tested experimentally to determine the effect of the modifications on the binding activity of different reader proteins as well as on the viral replication efficiency.

Comparing the different groups in the m^6^A machinery, the reader group contains more components than the writer and eraser groups, especially when taking into account the new readers recently discovered (Williams et al., 2019; Baquero-Perez et al., 2021). However, their roles have not been well studied, especially for the “direct” readers that lack an YTH domain. For example, HNRNPA2B was previously reported to be a direct reader, but its specific binding pattern with its m^6^A is poorly understood. Recently, structural and biochemical data have shown that this protein may actually be an “indirect” m^6^A reader, as studies have shown that HNRNPA2B1 does not specifically recognize m^6^A-labeled RNA *in vitro* and *in vivo* (Wu et al., 2018). In addition, the roles of the three YTH domain-containing readers DF1–3 in regulating viral replication are inconsistently reported. On the one hand, this may be due to the virus and cells used; on the other hand, although previous studies have indicated different functions of these readers, their functions cannot always be directly translated to viral RNAs. In other words, these readers may not always bind to the m^6^A sites at the same time or with maximum binding affinity. This speculation leads to further specific questions: since all YTH-reader paralogs share a conserved m^6^A binding motif, do they bind to the same copy of the RNA transcript? Do they compete each other for the same sites to have an antagonistic effect or do they bind at the same location to form a complex to cause a synergistic effect? These fundamental biological questions can be answered by assays using a designed luciferase reporter construct generated by site-directed mutagenesis of the specific m^6^A sites.

The importance and role of the epitranscriptome in regulating viral replication and antiviral immunity open the door for novel therapeutic interventions that target either the viral or host epitranscriptome to suppress viral replication. For viruses such as HMPV, HCMV, and VSV that take advantage of m^6^A methylation to escape the recognition of the host innate immune response, knockdown of the methyltransferases or reader proteins with siRNA or small-molecule inhibitors is a good antiviral strategy. In addition, generation of m^6^A-deficient viral strains might be useful when creating attenuated live viral vaccines because m^6^A methylation of viral RNA negatively regulates the host innate immune response and m^6^A-depleted virus induces a potent T1IFN response *in vivo* (Lu et al., 2020). Two good examples are the RSV G-transcripts depleted of m^6^A and the m^6^A-deficient HMPV virus, which are highly attenuated yet retain high immunogenicity in cotton rats (Xue et al., 2019; Lu et al., 2020). For viruses that are suppressed by m^6^A modifications, similar strategies can be used for drug and vaccine development, but the approaches would be to enhance m^6^A methylation with specific compounds (Selberg et al., 2019) and generate mutant virus with enhanced m^6^A methylation. Examples include flaviviruses and SARS-CoV2 RNA, whose genomes are gradually m^6^A-methylated during infection in host cells; these m^6^A additions negatively modulate the life cycle of these viruses, while knockdown of METTL3 and/or METTL14 dramatically increases viral particle formation (Gokhale et al., 2016; Lichinchi et al., 2016b; Liu et al., 2021a). These findings suggest that strategies to enhance the m^6^A methylation of viral RNA may be a potential therapeutic avenue to develop vaccines or antiviral drugs for these viruses. Although the functionality of such proposed vaccines has not been evaluated, it seems plausible that infection with live attenuated vaccines based on altered m^6^A methylation might induce potent adaptive immune responses providing the host with long lasting immunity to the wild-type virus.

Although vast advances have been made in recent years, our understanding of how m^6^A modification contributes to the regulation of gene expression and viral replication is still in its infancy. In particular, viral infection is a complex process of virus–host interaction, and epitranscriptomic analysis must expand its scope to include the m^6^A landscape of host transcripts. Most previous studies used cell lines, which limits investigations to the host immune responses underlying the mechanisms of disease induction. Future research should involve animal models to reveal the related pathogenesis of human diseases. The recently improved m^6^A sequencing techniques and CRISPR/Cas9 gene editing approaches will clearly be a major help for this endeavor.

## Author contributions

DY: writing original manuscript. GZ and HZ: revision and editing. All authors contributed to the article and approved the submitted version.
